# A Novel Cyclin-Dependent Kinase 13 Variant and Unusual Association of Situs Inversus Partialis in a Child From Bahrain: A Case Report and Literature Review

**DOI:** 10.7759/cureus.60970

**Published:** 2024-05-24

**Authors:** Hasan M Isa, Abdulla M Abdulla, Kawthar M Abdulla, Marwa J Abdulnabi, Zainab A Khudhair, Zakariya J Hubail, Maryam Y Busehail, Hasan A Abdulrasool

**Affiliations:** 1 Department of Pediatrics, Arabian Gulf University, Manama, BHR; 2 Department of Pediatrics, Salmaniya Medical Complex, Manama, BHR

**Keywords:** facial dysmorphisms, motor and intellectual delay, abdominal situs inversus, congenital heart defects, cdk13

## Abstract

Cyclin-dependent kinase 13 (CDK13)-related disorder is a rare autosomal dominant disease caused by pathogenic variants in the *CDK13* gene. This disorder was found to be related to several clinical features, including structural cardiac anomalies, developmental delay, anomalies of the corpus callosum, and a variety of facial dysmorphisms. In addition, feeding difficulties and neonatal hypotonia might also present. The diagnosis of this disorder is based on molecular genetic testing to detect the causative pathogenic variants. Here, we report a case of a one-year-old girl from Yemen, residing in Bahrain, with a CDK13-related disorder who was found to have an unusual association of abdominal situs inversus along with multiple structural cardiac anomalies, including atrial septal defect, ventricular septal defect, patent ductus arteriosus, interrupted inferior vena cava, bilateral superior vena cava, mild coarctation of the aorta, dilated coronary sinuses, and mild regurgitation in the tricuspid valve. Moreover, facial dysmorphism including medial epicanthal folds, posteriorly rotated ears, and a depressed nasal bridge was also noted. Further assessment showed a delay in reaching developmental milestones, including speech and motor delay. The patient also presented with recurrent episodes of upper respiratory tract infections, acute bronchiolitis, and lobar pneumonia which required admission to the intensive care unit and ventilation. The last infection episode was at the age of one year. Thereafter, the patient underwent cardiac repair of the ventricular septal defect followed by no more infection episodes until the age of one year and two months. The diagnosis of CDK13 was confirmed by a whole exome sequencing test which demonstrated a novel missense variant in exon 14 of the *CDK13* gene as a variant of uncertain significance in a heterozygous state.

## Introduction

Cyclin-dependent kinases (CDK) regulate the cell cycle and ribonucleic acid polymerase II transcription from initiation to elongation through phosphorylation of key residues in the C-terminal domain [1]. The structure of cyclin-dependent kinase 13 (CDK13) contains a C-terminal extension helix, which is a characteristic feature of transcription elongation kinases [1]. CDK13-related disorder is a rare hereditary autosomal dominant disorder [2]. The first seven individuals with CDK13-related disorder were recently reported, in 2016, by Sifrim et al. while studying a large cohort of patients with congenital heart diseases tested via exome sequencing [3]. Up to 2022, only 62 cases of this disease have been reported globally [4]. However, the prevalence of CDK13 worldwide is still unknown [2].

Despite the limited knowledge of CDK13-related disorder, several features were found to be related to this condition, including structural cardiac anomalies, developmental delay, anomalies of the corpus callosum, feeding difficulties, neonatal hypotonia, a variety of facial dysmorphisms such as hypertelorism, upslanting palpebral fissures, a wide nasal bridge, epicanthal folds, low-set ears, a small mouth with a thin upper lip vermilion, autism, and seizures [5-7]. This disease is also known as congenital heart defects, dysmorphic facial features, and intellectual developmental disorder (CHDFIDD) which reflects its clinical presentations [8]. Based on the variety of clinical presentations, this disorder is usually diagnosed by molecular genetic testing to detect the pathogenic variants in CDK13 [2].

In this report, we describe the case of CDK13-related disorder in a child from Yemen, residing in Bahrain, the first case in this region. In addition to the classical clinical presentations, our patient had an unusual association with an abdominal situs inversus. To our knowledge, this association was not previously reported in patients with CDK13-related disorders.

## Case presentation

**Figure 1:**
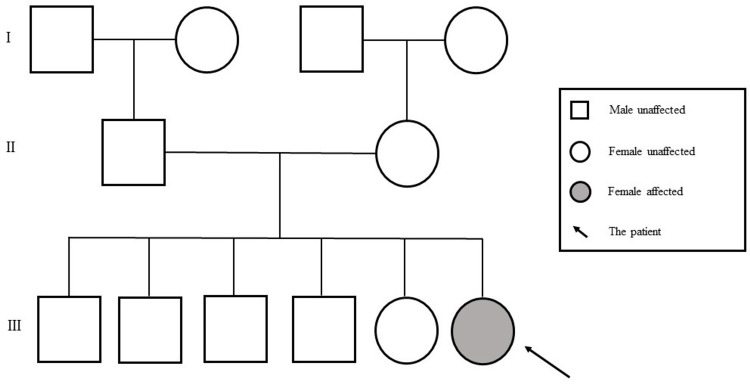
Family pedigree of the patient with cyclin-dependent kinase 13-related disorder. Image credits: Authors

This is the case of a 14-month-old female child from Yemen, residing in Bahrain, who was born at 38 weeks of gestation through a normal vaginal delivery. The patient is the sixth child of non-consanguineous parents. She has four brothers and one sister, all of whom were healthy (Figure 1).

The mother had diabetes mellitus and was on insulin for seven years before labor. During the third trimester, her vaginal swab was positive for group B *Streptococcus*. Therefore, she received intravenous antibiotics during labor. After delivery, the newborn’s Apgar score was 9, 10, and 10 at one, five, and 10 minutes, respectively. Her birth weight was 2.8 kg (16th percentile), her height was 49 cm (47th percentile), and her head circumference was 33 cm (23rd percentile). A small scalp wound was noted at the probe site, which was treated with pantothenic acid cream. She tolerated both breast and formula feeding with no history of any allergy.

Soon after birth, the patient was admitted to the neonatal intensive care unit due to cardiac concerns. She was pink, icteric, but not cyanosed. The anterior fontanelle was flat and widely opened. A grade 3 ejection systolic murmur was best heard on the left sternal border. Hypotonia and talipes equinovarus were also detected. Other systemic examinations were unremarkable. Screening blood sugar and thyroid stimulating hormone levels were normal. Laboratory findings of the patient are shown in Table 1.

**Table 1:** Laboratory results of the patient with cyclin-dependent kinase 13-related disorder. MCV, mean cell volume; MCH, mean cell hemoglobin; RBCs, red blood cells; MPV, mean platelet volume; WBCs, white blood cells; CRP, C-reactive protein; NR, no record; ALP, alkaline phosphatase; GGT, Gamma-glutamyl transpeptidase.

Laboratory test	Normal range	Patient age (weeks)						
		0.0	1.0	3.2	30.0	33.1	33.5	41.0
Hemoglobin (g/dL)	12.0-14.5	13.0	14.1	12.7	10.6	10.1	10.6	9.0
Hematocrit (%)	33-45	56.4	43.6	39.8	32.6	31.1	33.3	28.6
MCV (fL)	82-97	116.0	106.6	103.0	75.9	74.9	76.0	72.6
MCH (pg)	27-33	26.7	34.5	33.3	24.6	24.3	24.3	22.8
RBCs (cells/µL)	3.9-5.2	4.8	4.1	3.8	4.2	4.1	4.3	3.9
Platelets (×10⁹/L)	140-440	153	264	308	440	325	417	636
MPV (fL)	7.8-11.0	9.5	9.4	9.6	8.0	9.0	8.8	9.2
WBCs (×10⁹/L)	3.6-9.6	11.4	7.8	6.1	14.7	11.3	7.1	10.8
Neutrophils (%)	42.2-75.2	61.1	29.7	21.8	19.1	53.5	32.0	44.8
Lymphocytes (%)	20.5-51.1	30.3	55.0	61.9	70.9	41.0	64.4	46.0
Monocytes (%)	1.7-9.3	7.1	12.4	11.1	4.8	5.0	3.0	8.1
Eosinophils (%)	1-6	1.0	3.0	4.7	5.0	0.6	0.6	0.2
Basophils (%)	0-2	0.6	0.2	0.9	0.3	0.3	0.1	0.6
CRP (mg/L)	0-3	1.4	NR	NR	11.0	7.6	5.3	1.9
Total protein (g/L)	57-82	NR	54	52	NR	NR	NR	67
Albumin (g/L)	38-54	NR	35	35	NR	NR	NR	48
Globulin (g/L)	15-30	NR	19	17	NR	NR	NR	19
Total bilirubin (µmol)	<17	NR	201	67	NR	NR	NR	6
Direct bilirubin (µmol)	<5.1	NR	18	16	NR	NR	NR	NR
Indirect bilirubin (µmol)	<18	NR	183	51	NR	NR	NR	NR
ALP (U/L)	150-420	NR	249	424	NR	NR	NR	260
GGT (U/L)	<38	NR	77	112	NR	NR	NR	14

**Figure 2:**
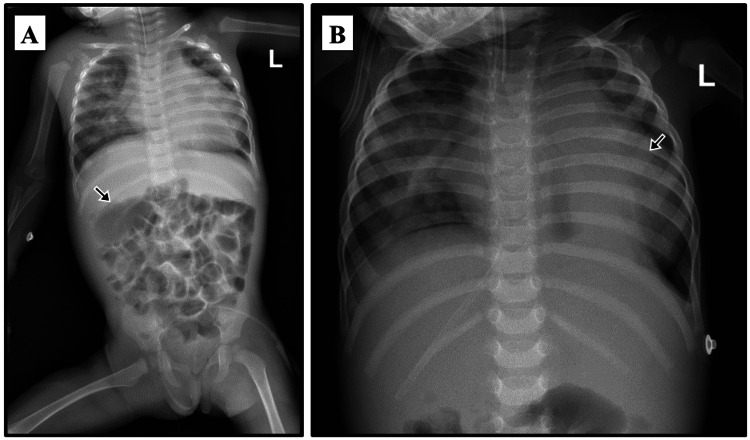
A plain X-ray of the chest and abdomen of the patient with cyclin-dependent kinase 13-related disorder. (A) The nasogastric tube (arrow) passes to the right side towards the right-sided stomach indicating the situs inversus partialis. (B) Right-sided lung infiltrate indicating pneumonia with an enlarged cardiac shadow indicating cardiomegaly (arrow).

Perianal sample culture grew extended-spectrum beta-lactamase *Escherichia coli* sensitive to gentamicin which was started intravenously in combination with ampicillin and continued for six days. Cerebrospinal fluid and blood cultures were sterile. Chest and abdominal radiographs showed an abdominal situs inversus (Figure 2A).

Abdominal ultrasound revealed that the stomach and spleen were seen on the right side, while the liver was in the midline and the gallbladder was partially distended. The skull ultrasound was unremarkable. An echocardiogram revealed an interrupted inferior vena cava (IVC), a patent ductus arteriosus (PDA), an atrial septal defect (ASD) with 2 mm secundum defect, a patent foramen ovale of 2 mm, a ventricular septal defect (VSD) of peri-membranous type measuring 7 mm, mild coarctation of the aorta, bilateral superior vena cava (SVC), dilated coronary sinuses, and mild regurgitation in the tricuspid valve. Since the patient was clinically stable, she was discharged home without medications.

At the age of 23 days, in the outpatient follow-up visit, the patient was active, pink, moving all limbs, feeding well, weighing 3.2 kg (7th percentile), and the scalp wound was healed. The heart murmur was changed to a pansystolic grade 3/6 murmur with an ejection click heard on the upper left sternal border, and the liver edge was 3 cm below the costal margin.

At the age of three months, in view of the patient’s cardiac defects, abdominal situs inversus, and poor overall growth, the geneticist requested a whole exome sequencing (WES) test to rule out primary ciliary dyskinesia.

At the age of five months, the genetic results demonstrated a novel missense variant in exon 14 of the *CDK13* gene. This was a heterozygous variant of uncertain significance which is associated with congenital heart defects, dysmorphic facial features, and intellectual development disorder.

Later, the patient experienced multiple hospital admissions. At the age of seven months, she was admitted with cough, runny nose, breathing difficulty, and decreased oral intake. She was tachypneic with nasal flaring, subcostal retractions, bilateral chest wheezes, and an ejection systolic murmur. She was diagnosed with acute bronchiolitis. C-reactive protein (CRP) was elevated (11 mg/L, normal range: 0-3) but the respiratory profile was negative. Chest x-ray showed bilateral hyperinflated lungs, while arterial blood gases were normal. The patient required oxygen (O2) via nasal cannula, nebulized salbutamol and adrenaline, and intravenous fluid infusion. Ceftriaxone was administered which was changed to amoxicillin-clavulanic acid on discharge after a week of admission. Two weeks later, she was hospitalized again with similar symptoms along with vomiting and diarrhea. She was in respiratory distress, had bilateral wheezes and crepitations, and had a pansystolic murmur. Chest x-ray showed bilateral air bronchogram with right-sided patchy infiltration and cardiomegaly (Figure 2B).

She was diagnosed with pneumonia on top of acute bronchiolitis. Blood culture grew *Micrococcus luteus* which required ceftriaxone along with O2 and bronchodilators. Three days later, repeated blood culture grew a coagulase-negative Staphylococci epidermidis methicillin-resistant strain. Due to her cardiac condition, furosemide was administered. The patient was stabilized and discharged home. 

**Figure 3:**
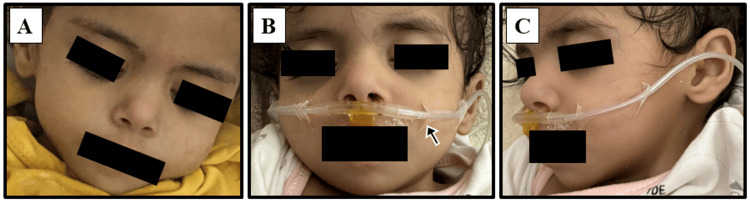
The patient, receiving oxygen via nasal cannula (arrow), with dysmorphic features including epicanthal folds (A and B), posteriorly rotated ears (B and C), and micrognathia (C)

At the age of eight months, at the pediatric cardiology clinic, the patient had an upper respiratory tract infection. She weighed 5.1 kg (<1st percentile), her length was 61 cm (<1st percentile), and her head circumference was 41 cm (4th percentile). She was thin with facial dysmorphic features including epicanthal folds, a depressed nasal bridge, and posteriorly rotated ears (Figure 3).

Her examination revealed an increased precordial activity, a cardiac thrill, and a pansystolic murmur. Echocardiography revealed a dilated left atrium and ventricle, a VSD measuring 3.5 mm, restrictive left to right shunting, pulmonary stenosis with a pressure reading of 40 mmHg, a dilated main pulmonary artery measuring 24 mmHg and a flow reversal, a small aortic arch with the narrowest part of the lumen measuring 3 mm, and a peak instantaneous doppler pressure of 16 mmHg. In addition, the patient had bilateral SVC and a dilated coronary sinus. A surgical repair of cardiac defects was planned.

At the age of nine months, she had shortness of breath with peripheral O2 saturation (SpO2) of 89-90%, dry cough, and a congested nose along with reduced oral intake, activity, and urine output. She had sunken eyes and anterior fontanelle, tachypnea with nasal flaring, chest retractions, bilateral wheezes, a palpable liver along with the previously heard heart murmur. Acute bronchiolitis versus pulmonary congestion was the diagnosis as she was not receiving furosemide regularly. The N-terminal brain natriuretic peptide (BNP) level was >35,000 ng/L (normal range <125), and the troponin-I level was 1.9 ng/mL (normal range <1.5). Blood gas analysis showed mild respiratory acidosis. Chest x-ray and electrocardiogram were unremarkable. Subsequently, intravenous fluid increased gradually, and furosemide was restarted once the urine output was acceptable. Ceftriaxone was also administered. After clinical improvement, she was discharged home on oral furosemide. However, 10 days later, the patient was readmitted with similar symptoms and was managed symptomatically.

At the age of 11 months, she presented with irritability, tachypnea, intermittent nocturnal cough, shortness of breath, chest retractions, and decreased appetite. The patient was kept on O2 via face mask along with furosemide. Her SpO2 dropped to 78%, with a respiratory rate of 62 breaths per minute and a heart rate of 180 beats per minute. She was intubated and transferred to the pediatric intensive care unit (PICU) where she was diagnosed with coronavirus disease 2019 (COVID-19)-related multisystem inflammatory syndrome of children (MIS-C) with myocarditis and pneumonia. Intravenous immunoglobulins and methylprednisolone were administered followed by oral dexamethasone for eight days. Blood culture grew *Staphylococcus hominis* and she was kept on clindamycin and ceftriaxone for seven days.

The patient was transferred to the general ward, extubated, and placed on airway-humidified high-flow therapy (AIRVO). However, she was reintubated on the same day as she became cyanosed with respiratory distress and subcostal retractions. Her SpO2 was 50%, her heart rate was 120 beats per minute, and her blood pressure was 87/65 mmHg. After reintubation, fentanyl infusion, and furosemide, SpO2 improved to 100%. Yet, the air entry was reduced on the left side with bilateral crepitations. BNP level was still >35,000 ng/L, troponin increased to 48.870 ng/mL, and alanine aminotransferase increased from the normal level to 1663 U/L (normal range <33). The patient stayed in the PICU for another eight days, where midazolam, budesonide, spironolactone, omeprazole, and piperacillin/tazobactam were also administered. After stabilization, she was transferred to the general ward and then discharged home. Three days later, she was admitted again due to respiratory distress and SpO2 of 88%. She was also tachypneic with chest retractions, bilateral crepitations, and wheezes. CRP level was high (44 mg/L), the BNP level declined to 4472 ng/L, and the troponin-I level was normalized. Chest x-ray showed bilateral haziness. The patient was treated with O2 via nasal cannula, intravenous fluid, ceftriaxone, and furosemide. Later, ceftriaxone was changed to cefepime and clindamycin. After improvement, she was discharged home on oral furosemide.

At the age of one year, the patient underwent surgical closure of the VSD. Currently, the patient is one year and two months old. She is stable with no further infections noted after the surgery. She is receiving tapering doses of furosemide, enalapril, and spironolactone. Her current weight is 7.5 kg (3rd percentile) and her height is 70.5 cm (1st percentile). She has regular follow-up appointments scheduled with the cardiologist and dietitian in view of her cardiac condition and growth faltering.

## Discussion

This study described the first case of CDK13-related disorder diagnosed in a female infant from Yemen, residing in Bahrain. CDK13-related disorder is a rare autosomal dominant disease, therefore, there is no gender preference for this disease [2]. Nonetheless, most of the previously published cases were females (n=39/62, 62.9%) (Table 2) [4].

**Table 2:** Clinical characteristics of patients with cyclin-dependent kinase 13-related disorder as reported in the literature. F, female; M, male; NR, no record.

Study	Number of patients	Number as per gender	Congenital heart diseases	Dysmorphic features	Intellectual and developmental disabilities	Recurrent infection
Sifrim et al., 2016 [3]	7	5F, 2M	7/7 (100%)	7/7 (100%)	7/7 (100%)	NR
Rouxel et al., 2022 [4]	18	10F, 8M	12/18 (66.7%)	18/18 (100%)	16/17 (94.1%)	6/18 (33.3%)
Hamilton et al., 2017 [5]	16	14F, 2M	9/16 (56%)	16/16 (100%)	16/16 (100%)	3/16 (18.7%)
Tomoko et al., 2017 [6]	3	1F, 2M	0/3 (0%)	3/3 (100%)	3/3 (100%)	NR
Van et al., 2018 [7]	15	9F, 6M	2/13 (15%)	15/15 (100%)	15/15 (100%)	NR
Bostwick et al., 2017 [8]	16	9F, 7M	13/16 (81%)	13/13 (100%)	16/16 (100%)	NR

Cardiac anomalies are known to be associated with CDK13-related disorder [6]. The prevalence of congenital heart disease (CHD) is around 46% [2]. ASD and VSD, followed by pulmonary valve abnormalities, are the most common structural cardiac anomalies found to be associated with CDK13 [2,8,9]. Our patient was found to have multiple congenital heart defects, including ASD, VSD, PDA, interrupted IVC, bilateral SVC, mild coarctation of the aorta, dilated coronary sinuses, and mild regurgitation in the tricuspid valve. Similarly, all the first seven described cases had congenital heart defects [8]. However, Hamilton et al. reported seven patients with CDK13-related disorder without congenital heart defects [5]. This was also the case in the studies by Uehara et al. and Bostwick et al. which reported three patients each [6,8]. Therefore, although CHD is a prevalent feature of this disease, it is not a mandatory defining feature.

Our patient was found to have abdominal situs inversus with levocardia (situs inversus partialis). Upon extensive literature review, we could not find any documented patients with CDK13-related disorder and abdominal situs inversus. This distinct association represents a novel finding that has not been reported previously.

Characteristics of facial features, including hypertelorism, epicanthal folds, flat midface, wide nasal bridge, short columella, small mouth, low-set ears, and highly arched eyebrows were also reported in patients with CDK13 disorder [5,8]. Our patient also had medial epicanthal folds, a depressed nasal bridge, and posteriorly rotated ears.

Upon literature review, it was seen that almost all reported cases have motor and language delays [4,7,8]. Van den Akker et al. stated that all of their patients showed variable degrees of developmental delays and intellectual disabilities [7]. Similarly, our patient showed a motor and speech development delay along with growth faltering.

Our patient had several admissions to the hospital due to recurrent attacks of upper respiratory tract infection, acute bronchiolitis, pneumonia, and one episode of acute gastroenteritis. Similarly, Rouxel et al. noted that 33.3% of the patients had recurrent infections [4]. Hamilton et al. also reported recurrent gastrointestinal, ear, and upper respiratory tract infections in their patients [5]. The reason for recurrent infections in patients with CDK13-related disorder has not been sufficiently discussed. Berro et al. proposed that the *CDK13* gene might play a role in immunity while studying human immunodeficiency virus type 1, and they described that CDK13 might act as a virus-regulating factor [10]. However, this explanation needs further exploration.

In view of the variety of clinical presentations, the diagnosis of CDK13-related disorder is based on genetic testing [2]. CDK13 is an autosomal dominant disorder and only one allele of a mutated gene is necessary for the disease to be present [2]. It is caused by a de novo CDK13 pathogenic variant [2]. The diagnosis of CDK13 in our patient was confirmed by a WES test which demonstrated a novel missense variant in exon 14 of the *CDK13* gene, as a heterozygous variant of uncertain significance, that results in the amino acid substitution of threonine for alanine at codon 1435 (p.Ala1435Thr; ENST00000181839). This variant has not been reported in the 1000 genomes, gnomAD (v3.1), gnomAD (v2.1), and Trans-Omics for Precision Medicine (TOPMed) databases and has a minor allele frequency of 0.001% in the MedGemone Labs internal database (MedGenome Labs Private Ltd., Chennai, India). The reference codon is conserved across species. According to Hamilton and Suri's study, the most documented cases had missense mutations (n=36/44) while the minority had splice site (n=3/44), recurrent frameshift (n=3/44), or nonsense mutations (n=2/44) [9].

The management of patients with CDK13-related disorder depends mainly on symptomatic treatment, including the use of oxygen therapy, bronchodilators, diuretics, and antibiotics. Cardiac and neurological evaluations of patients diagnosed with CDK13 are recommended [7,9]. In our patient, in addition to cardiac evaluation which revealed the presence of multiple CHDs, a cranial ultrasound was performed which excluded obvious brain abnormalities. However, Bostwick et al. reported central nervous system anomalies in 10 of their cases (n=10/11, 91%) [8].

## Conclusions

CDK13-related disorder is a rare autosomal dominant disease characterized by dysmorphic facial features and intellectual developmental disorder with or without congenital heart disease. In this report, we described the first patient with this disease from Bahrain, a one-year-old Yemeni girl, who was found to have an unusual association of abdominal situs inversus besides the multiple structural cardiac anomalies, facial dysmorphism, and recurrent chest infections. Moreover, a novel *CDK13* gene variant was detected. Further research is needed to understand the full spectrum of the disease's clinical presentations and the potential long-term outcome.
